# Editorial: Proceedings of the “Fourth International Conference of the *FMR1* Premutation: Basic Mechanisms, Clinical Involvement and Therapy”

**DOI:** 10.3389/fmolb.2021.671875

**Published:** 2021-04-27

**Authors:** Karen Usdin, Laia Rodriguez-Revenga, Rob Willemsen, Renate Hukema, Cecilia Giulivi

**Affiliations:** ^1^Gene Structure and Disease Section, Laboratory of Cell and Molecular Biology, National Institute of Diabetes, Digestive and Kidney Diseases, National Institutes of Health, Bethesda, MD, United States; ^2^Biochemistry and Molecular Genetics Department, Hospital Clinic of Barcelona and CIBER of Rare Diseases (CIBERER), Instituto de Salud Carlos III, Barcelona, Spain; ^3^Department of Clinical Genetics, Erasmus MC, Rotterdam, Netherlands; ^4^Department of Molecular Biosciences, School of Veterinary Medicine, University of California, Davis, Davis, CA, United States; ^5^The MIND Institute, University of California, Davis Medical Center, Sacramento, CA, United States

**Keywords:** *FMR1* premutation, triplet nucleotide repeats, AGG interruptions, FXTAS, FXPOI, mouse model, mitochondrial dysfunction

*Fragile X mental retardation 1* (*FMR1)* is an X-linked gene with a hypervariable CGG repeat tract in its 5′ UTR. Alleles with 55–200 CGG repeats are known as premutation alleles (Kogan et al., 2008). Alleles with >200 repeats, known as full mutation alleles, are responsible for Fragile X syndrome, the most common inherited form of intellectual disability and autism spectrum disorder (Verkerk et al., 1991; Yu et al., 1991). The repeat tract in premutation alleles expands on intergenerational transmission to produce larger premutation or full mutation alleles. However, the mechanism responsible for this expansion is largely unknown. Premutation alleles, once thought to be asymptomatic, are now known to confer risk of developing Fragile X-associated tremor/ataxia syndrome (FXTAS), a neurodegenerative disorder (Jacquemont et al., 2003; Grigsby et al., 2006) and a form of ovarian dysfunction known as Fragile X-associated primary ovarian insufficiency (Sherman, 2000; Wittenberger et al., 2007). Premutation carriers can also have other symptoms including fibromyalgia, chronic fatigue, and sleep problems that are referred to collectively as the Fragile X-Associated Neuropsychiatric Disorders (Hagerman et al., 2018). The molecular basis of the pathology seen in premutation carriers is still the subject of much debate (Glineburg et al., 2018).

This special collection of *Frontiers in Cell and Developmental Biology* contains contributions from leading groups in this field that were presented at the 4th International Conference of the *FMR1* Premutation held in Rotterdam on September 25–27, 2019 organized by Drs. Renate Hukema and Rob Willemsen. This collection covers recent advances in basic and clinical research into the underlying mutation and pathology associated with the *FMR1* premutation.

## Mechanisms of Expansion and Disease Pathology

Gazy et al. showed that embryonic stem cells from *FMR1* premutation mice (Entezam et al., 2007) recapitulate expansion in a dish, thus providing the first cell model that can be used to study expansion mechanisms. As in cells from human premutation carriers, these cells show mitochondrial abnormalities (Napoli et al., 2016), an *FMR1*-associated R-loop (Groh et al., 2014; Loomis et al., 2014; Kumari and Usdin, 2016) and elevated *Fmr1* transcription (Tassone et al., 2000). Interestingly, increased transcription was sensitive to O_2_ tensions. Since these cells can be readily gene-edited, they may help expedite studies of the expansion mechanism as well as premutation pathology.

One model for premutation pathology proposes that RAN translation from premutation alleles produces toxic proteins including FMRpolyGlycine (FMRpolyG), that are responsible both for disease pathology and the intranuclear neuronal inclusions that are a hallmark of FXTAS (Krans et al., 2019). Two papers in this issue address the issue of the role of FMRpolyG. Haify et al. describes an inducible mouse model of FXTAS containing the 5' UTR of the human *FMR1* gene with 103 CGG repeats cloned downstream of a dox-inducible *CamKII*-α promoter. This allowed the regulated expression of FMRpolyG by doxycycline. However, after 12 weeks of FMRpolyG induction no evidence of a behavioral phenotype was seen despite the presence of many intranuclear inclusions throughout the brain. Thus, neither inclusions nor the expression of FMRpolyG *per se* are sufficient to cause neurological problems, at least in young adult mice.

Holm et al. describe the analysis of the cerebral cortex proteome in individuals with FXTAS. Significant differences from the normal proteome were observed including decreased tenascin-C (TNC) and increased levels of the small ubiquitin-like modifier 1/2 (SUMO1/2). FMRpolyG, which has only been identified in trace amounts in studies of FXTAS inclusions (Ma et al., 2019), was not identified in either FXTAS or control brains. Interestingly, in contrast to many other neurodegenerative diseases, the proteome of end-stage FXTAS provides no evidence for a strong inflammation-mediated degenerative response.

## Clinical Involvement in *FMR1* Premutation Carriers

Johnson et al. report on the recommendations of the European Fragile X Network, made in consultation with other stakeholders at the meeting, that the term Fragile X Premutation Associated Conditions (FXPAC) be used to encompass all conditions related to the premutation. This recommendation was made in part to avoid stigmatization of carriers and to facilitate patient evaluation and treatment.

Two papers address metabolic alterations in premutation carriers. Cao et al. review the altered metabolites identified in previous studies of plasma from premutation carriers and the cerebella of FXTAS mice. Napoli et al. address the metabolic footprint of plasma from female carriers using a combined multi-omics approach. Down-regulation of RNA and mRNA metabolism, protein translation, carbon and protein metabolism and the unfolded protein response, and up-regulation of glycolysis and the antioxidant response were observed. Some changes were linked to decreased protein translation, but others seemed to be secondary to oxidative stress.

Finally, Tassanakijpanich et al. discuss rarely appreciated cardiovascular problems in premutation carriers and possible contributing mechanisms including RNA toxicity and mild FMRP deficiency. The review underscores cardiac arrhythmia, autonomic dysfunction, and hypertension as problems that clinicians need to be aware of in this population.

## Motor and Neurocognitive Profile of *FMR1* Premutation Carriers

Although FXTAS is more prevalent and severe in males than females, specific sex differences have not been well-documented. In this section, Loesch et al. report a 2-fold faster progression in males than in females in key measures of tremor and ataxia, while psychiatric symptoms only progressed in females. They postulate the existence of neuroprotective effects beyond the presence of one normal *FMR1* allele in female carriers, specifically affecting cerebellar circuitry.

Winston et al. describe patterns of visual attention in premutation carriers, parents of individuals with autism spectrum disorders, and typically developing controls. Their results demonstrate a visual attention profile that appears strongly associated with the premutation in women and that thus may constitute a meaningful biomarker. Mailick et al. compared the response to parenting stress in mothers with “low zone” (LZ; ≤ 25 CGGs) alleles to mothers whose repeats were in the normal range. LZ mothers who had children with disabilities had greater limitations in executive functioning, depression, anxiety, daily health symptoms, and balance, than LZ mothers of non-disabled children. In contrast, mothers with normal-range CGG repeats did not differ based on stress exposure consistent with greater resilience.

## Analysis of CGG Repeat Alleles

Villate et al. report the analysis of 87 maternal transmissions of alleles with 45–65 repeats and variable numbers of AGG interruptions. Their results confirm the protective effect of AGGs reported previously (Nolin et al., 2013). The authors suggest that assessment of the risk of unstable transmissions should be based on the presence or absence of AGG interruptions and not on the classical cutoffs that define different *FMR1* alleles. Rodrigues et al. described a new approach for evaluating normal *FMR1* alleles that includes the contribution from the AGG interspersion pattern of each allele. The outcome, a numerical parameter named “*allelic score,”* describes the allelic complexity of the *FMR1* gene and provides an additional tool to evaluate pathogenicity and expansion risk.

## Concluding Remarks and Future Directions

The studies published in this special collection demonstrate just how far our understanding of the pathogenic mechanisms, disease diagnosis and management of affected individuals has come in the few years since the 3rd International Meeting on the *FMR1* Premutation. However, these studies also highlight the work that still needs to be done to improve our understanding and treatment of these disorders. Hopefully, some of these issues will be addressed at the 5th iteration of this conference scheduled for New Zealand in March 2022.

## Author Contributions

All authors listed have made a substantial, direct and intellectual contribution to the work, and approved it for publication.

## Conflict of Interest

The authors declare that the research was conducted in the absence of any commercial or financial relationships that could be construed as a potential conflict of interest.
